# Reality Check 2: The Cost-Effectiveness of Policy Disallowing Body Checking in Non-Elite 13- to 14-Year-Old Ice Hockey Players

**DOI:** 10.3390/ijerph18126322

**Published:** 2021-06-11

**Authors:** Gillian R. Currie, Raymond Lee, Luz Palacios-Derflingher, Brent Hagel, Amanda M. Black, Shelina Babul, Martin Mrazik, Deborah A. Marshall, Carolyn A. Emery

**Affiliations:** 1 Department of Paediatrics, Cumming School of Medicine, University of Calgary, Calgary, AB T3B 6A8, Canada; brent.hagel@ahs.ca (B.H.); caemery@ucalgary.ca (C.A.E.); 2 Department of Community Health Sciences, Cumming School of Medicine, University of Calgary, Calgary, AB T2N 4Z6, Canada; raymond.lee47@gmail.com (R.L.); damarsha@ucalgary.ca (D.A.M.); 3 Sport Injury Prevention Research Centre, Faculty of Kinesiology, University of Calgary, Calgary, AB T2N 1N4, Canada; lmpalaci@ucalgary.ca (L.P.-D.); ablack@ucalgary.ca (A.M.B.); 4 Department of Pediatrics, University of British Columbia, Vancouver, BC V6H 3V4, Canada; sbabul@bcchr.ca; 5 BCIRPU, BC Children’s Hospital, Vancouver, BC V6H 3V4, Canada; 6 Department of Educational Psychology, University of Alberta, Edmonton, AB T6G 2G5, Canada; mrazik@ualberta.ca; 7 McCaig Bone and Joint Health Institute, Calgary, AB T2N 4Z6, Canada

**Keywords:** economic evaluation, injury prevention, body checking policy, hockey, youth

## Abstract

Sport-related injuries are the leading cause of injury in youth and are costly to the healthcare system. When body checking is disallowed in non-elite levels of Bantam (ages 13–14 years) ice hockey, the injury rate is reduced, however the impact on costs is unknown. This study compared rates of game injuries and costs among non-elite Bantam ice hockey leagues that disallow body checking to those that did not. Methods: An economic evaluation was conducted alongside a prospective cohort study comparing 608 players from leagues where body checking was allowed in games (Calgary/Edmonton 2014–2015, Edmonton 2015–2016) with 396 players from leagues where it was not allowed in games (Vancouver, Kelowna 2014–2015, Calgary in 2015–2016). The effectiveness measure was the rate of game injuries per 1000 player-hours. Costs were estimated based on associated healthcare use within the publicly funded healthcare system as well as privately paid healthcare costs. Probabilistic sensitivity analysis was conducted using bootstrapping. Results: Disallowing body checking reduced the rate of injuries by 3.02 per 1000 player hours (95% CI −4.01, −1.35) and reduced public and total healthcare system costs by $ 1084 (95% CI $ −1716, $ −416) and $ 1100 (95% CI $ −1804, $ −346 per 1000 player-hours, respectively. These findings were robust in over 99% of iterations in sensitivity analyses in the public healthcare and the total healthcare system perspectives. There was no statistically significant difference in privately paid healthcare costs ($ −46 per 1000 player hours (95% CI $ −156, $ 70)). Interpretation: Disallowing body checking in non-elite 13–14-year-old ice hockey nationally would prevent injuries and reduce public healthcare costs.

## 1. Introduction

In Canada since 2010, over 600,000 youth participate each year in Hockey Canada youth ice hockey leagues [1]. Participating in a team sport has a range of benefits such as improving self-esteem, performance in academic studies, and mental health [2,3,4]. However, concussions and musculoskeletal injuries are a risk in hockey [5]. A national study of sport-related injury admissions to the emergency department found that ice hockey accounted for the highest proportion of concussions and musculoskeletal injuries compared with other sports in Canadians between the ages 5 and 19 years [6]. These injuries can cause permanent and detrimental effects that are seen later in life such as osteoarthritis [7,8,9]. There is also growing evidence that 13.7% of all children with concussions remain symptomatic longer than three months post-injury [10,11].

Body checking has been identified as the most consistent risk factor for all hockey-related injuries and concussions [12]. Body checking is a tactic used to gain an advantage on the opponent with the use of the body and occurs when a player intentionally plays the body of the opponent; often applying force in different direction for the purpose of stopping an attack or separating the opponent from the puck [13]. Hockey Canada introduced a national policy in 2013 disallowing body checking in all levels of play for 11–12-year-old players (i.e., Pee Wee level), which led to a 50% reduction in injury rates and a 64% reduction in concussion rates in hockey games [14]. In the absence of a national policy for 13–17-year-old levels, body checking policies are regulated in both municipal and provincial hockey associations in Canada, allowing natural comparisons between different policies [15]. A previous study conducted an economic evaluation of body checking policies in Pee Wee (age 11–12 years) hockey levels [16]. Healthcare costs and injury rates were 2.96- and 2.84-fold higher when body checking was allowed during games. It was estimated that 1273 injuries could have been prevented and $213,280 (2009 CAD) in healthcare costs could have been saved over one season if body checking was disallowed in Pee Wee hockey games in Alberta [16]. A similar policy in Bantam (age 13–14) hockey also reduced injury rates by 55% [17]; however, the effect on costs has not been studied. There has been a call for embracing the value of cost-effectiveness evidence in decision making and even making the use of cost-effectiveness analysis mandatory in clinical effectiveness research [18,19]. Evidence is needed on the associated costs to families and the healthcare system as a result of different rates of injuries from body checking to provide evidence and inform decisions on body checking polices. The objective of this study was to compare injury rates and costs between leagues with policies that allow or disallow body checking in non-elite 13- to 14-year-old hockey players.

## 2. Materials and Methods

An economic evaluation was conducted comparing the rates of game injuries and injury-related costs in a prospective cohort study of 13- to 14-year-old hockey players in British Columbia and Alberta. We used the Consolidated Health Economic Evaluation Reporting Standard (CHEERS) reporting guidelines [20].

Local ice hockey organizations were approached to recruit 13- to 14-year-old hockey players in the lower 60% level of play. Players from both Calgary and Edmonton in 2014–2015 and Edmonton in 2015–2016 comprised the cohort where body checking was allowed. Players from Kelowna and Vancouver in 2014–2015 and from Calgary in 2015–2016 comprised the cohort where body checking was disallowed. Full details of the cohort design, recruitment and data collection have been published previously [17]. The economic evaluation had a one-year time horizon including injuries and healthcare costs incurred by participants up to one year post-injury, and the base case analysis was from the public healthcare system perspective. Other scenarios included the private healthcare system perspective, as well as the total public and private combined.

### 2.1. Effectiveness

The measure of effectiveness used for the base case in this economic evaluation was rates of game injuries per 1000 player-hours to standardize the comparison between cohorts.

### 2.2. Healthcare Resource Use and Costs

Healthcare system resource use as well as private healthcare costs data were collected and included the frequency of healthcare professional visits, diagnostic imaging, medical treatments, and medication. These data were self-reported by the player or parent and recorded by a team designate on an injury report form (IRF) which also included weekly exposure to hockey sessions and injuries. Details of the treatment of missing data on exposure and injury variables were previously published [17]. For the healthcare utilization data, if it was indicated that a visit/test occurred but the number of tests/visits was missing, we made the conservative assumption that there was one visit. Table 1 shows the items that were included in each of the public and private healthcare system perspectives. Note that in the Canadian healthcare system, visits for healthcare professionals other than physicians are not covered by the publicly funded healthcare system.

### 2.3. Unit Costs

Unit cost sources from Alberta were used to standardize the costs when comparing policies and remove interprovincial variability. This included physician fee-for-service schedules [21] for general practitioners, sports medicine physicians, paediatricians, neurologists, emergency physicians, orthopaedic surgeons, and radiologists. The Alberta Ambulatory Care Classification System [22] was used to value emergency department visits and x-rays, ultrasounds, MRIs, and CT Scans. Unit costs for prescribed medication were from Alberta Blue Cross Drug Benefit List [23]. Unit costs for out-of-pocket healthcare use of physiotherapy, chiropractors, massage therapists, athletic therapists, braces, splints, tape, crutches, tensors, and over-the-counter medication were publicly available from vendors in Calgary. All costs were in Canadian dollars and converted to 2017 dollars. Healthcare costs were converted into costs per 1000 player-hours to standardize the comparisons between body checking policies.

### 2.4. Cost-Effectiveness Analysis

The differences in effectiveness and costs between the policy disallowing body checking and the comparator policy allowing body checking were calculated as the rate of injury or cost per 1000 player-hours, respectively, for the no body checking minus the body checking group. The cost-effectiveness analysis then jointly considered the cost and effectiveness differences. If disallowing body checking resulted in both a reduced rate of injury and a reduced cost, then this policy would be recommended. In scenarios where there were fewer injuries but higher costs or more injuries and lower costs, an incremental cost-effectiveness ratio (ICER) would be calculated (i.e., the ratio of the difference in cost per 1000 player-hours and the difference in the injury rate per 1000 player-hours). In that case, the ICER quantifies the trade-offs between costs and injury rates to be considered when making policy decisions.

### 2.5. Provincial and National Projection

The estimates from the cost-effectiveness analysis and data on average player-game hours were used to project the change in total injuries and total costs to all non-elite Bantam players registered in Alberta and also in Canada in the 2016–2017 season. The average game-hours estimate is 38.75 hours per Bantam player [17]. The population of Bantam players in Alberta 2016–2017 season was 7435, so the non-elite population (lower 60%) was 4461, and population in Canada was 63,587, so the non-elite (lower 60%) population was 38,152 [1].

### 2.6. Statistical Analysis

All data analyses were conducted in Stata v14 and R. Injury and cost rates for each group were calculated accounting for player game exposure hours. Non-parametric probabilistic sensitivity analysis was performed using bootstrapping with 10,000 iterations of sampling with replacement while first accounting for stratification by body checking cohort, and then resampling teams and subsequently participants and presented on an incremental cost-effectiveness plane scatterplot. Highest density bootstrap confidence intervals (95%) for the injury and costs rates for each group were calculated. As no specific R package for bootstrapping incorporates all these elements, our statistician adapted commands from several R functions including “draw.bootstrap”, “boot” and “resample”. For the provincial and national projections, confidence intervals were calculated from the injury and cost rate confidence intervals using the average game-hours and relevant population estimates.

## 3. Results

As previously reported [17], a total of 82 teams comprising 944 unique players were recruited in the study in the 2014–2015 and 2015–2016 seasons; 60 players participated in both seasons. There were 33 teams that were disallowed from body checking and 49 teams that were allowed to body check. As shown in Table 2, players disallowed from body checking had a total of 12,393 game participation hours in 396 players and 23,374 participation hours were observed in 608 players who were allowed to body check. When body checking was allowed, 129 injuries occurred compared with 31 injuries when body checking was disallowed. Table 3 shows that the distribution of baseline characteristics (sex, weight, height, player position, previous injury over the last year, previous concussion) was similar between the two groups [17].

Public and private healthcare resource use and costs (unadjusted for the difference in players or exposure hours) are presented in Table 4. Public healthcare costs accounted for 92% of total costs for the body checking group compared to 83% for the no body checking group. Within public healthcare spending, visits accounted for the majority of that category in both groups (63% body checking, 66% no body checking group). Within private spending, 86% of the private health costs are visit costs for the body checking group compared to 62% in the no body checking group. However, there were costs for ambulance visits only in the no body checking group and if these are not considered, visits account for 81% in that group.

### 3.1. Cost-Effectiveness Analysis Results

As shown in Table 5, for the base-case analysis using the public healthcare system perspective, there is a reduction in the rate of injuries by 3.02 per 1000 player hours (95% CI –4.01, −1.35) and a reduction in the costs by $ 1084 per 1000 player hours (95% CI $ −1716, $ −416) when body checking was disallowed compared with when it is allowed. There was no statistically significant difference in private healthcare costs ($ −46 per 1000 player hours (95% CI $ −156, $ 70)). Considering the total healthcare cost perspective, there was a reduction in costs by $ 1100 per 1000 player hours (95% CI $ −1804, $ −346).

**Figure 1:**
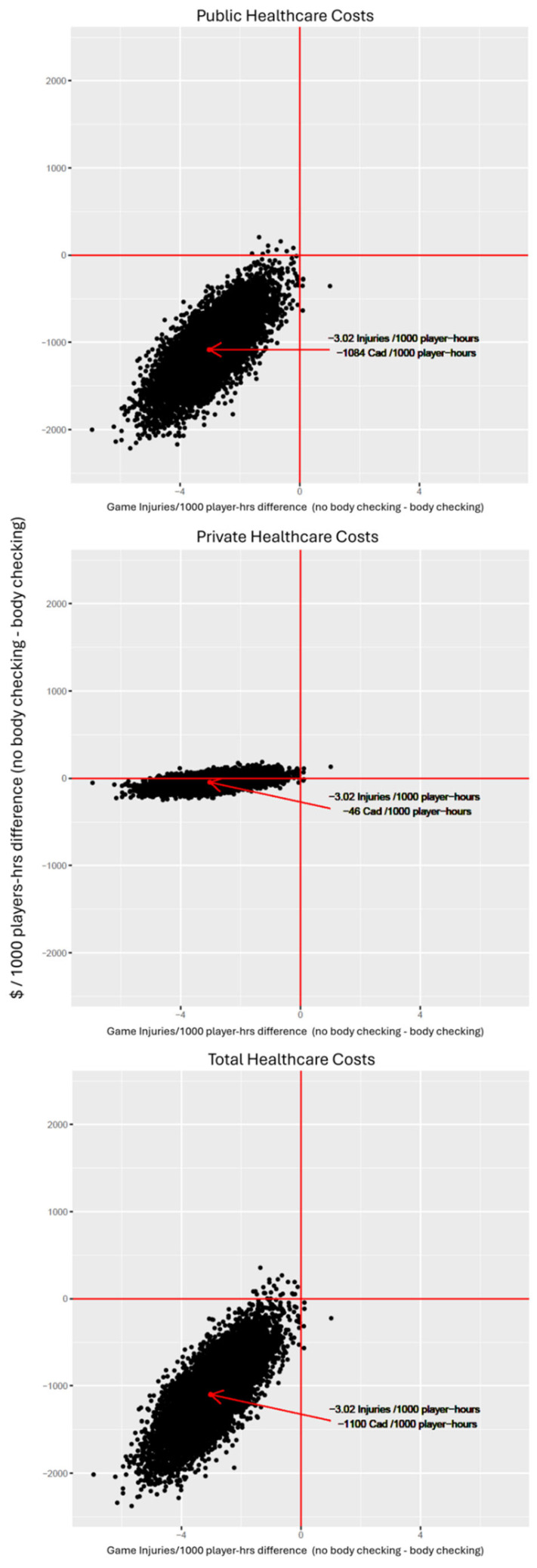
Probabilistic Sensitivity Analysis for policy comparison of body checking to no body checking.

In probabilistic sensitivity analysis (Figure 1), the no body checking policy reduces both injuries and costs from the public and total healthcare perspectives in 99.9% and 99.5% of iterations, respectively. Less than 1% of iterations fell within the other three quadrants for those two perspectives. For the private healthcare system perspective, although the point estimate difference was not statistically significant, 78% of the iterations fell within the quadrant where costs were lower and injuries were lower. For that perspective, 22% of iterations fell within the quadrant where costs were lower but injuries were higher and less than 1% of iterations fell within the other two quadrants.

### 3.2. Provincial and National Projection Results

As previously reported [17], if a policy disallowing body checking had been adopted in the 2015–2016 season, 522 injuries could have been prevented (95% CI −694, −233) in Alberta and 4461 injuries could have been prevented in Canada (95% CI −5932, −1994). In the current study, we similarly apply the cost rate reductions to the provincial and national player populations (see Table 6) to estimate a total potential savings of $ 187,364 public healthcare costs in Alberta (95% CI $ −296,617, $ −71,903) and $ 1,602,397 public healthcare costs in Canada (95% CI $ −2,536,769, $ −614,936). The results for the private healthcare cost and total healthcare cost perspective are presented in Table 6.

## 4. Discussion

### 4.1. Interpretation

These study findings support a policy recommendation to disallow body checking in games in 13- to 14-year-old ice hockey leagues as the policy is associated with both reduced rate of game-related injuries and reduced public healthcare costs. The findings were robust in sensitivity analysis. Projecting the results to all Bantam players in Alberta and Canada in the 2015–2016 hockey season, disallowing body checking in non-elite Bantam players could prevent 522 injuries and save $ 187,364 in public healthcare costs in Alberta. At a national level, this could prevent 4461 injuries and save $ 1,602,397 in public healthcare costs. In the context of total public healthcare spending in Alberta and in Canada (that is $ 21 and $ 161 billion, respectively in 2015 [24]), this is a small proportion; however, it is still an expenditure that could be saved, as well as avoiding injuries for these children. This study looks at injuries and costs over the one year of the study, so does not account for the longer-term impacts of those injuries.

These findings are consistent with previous economic evaluations of body checking policy in Pee-Wee ice hockey where injuries were lower and costs were lower or remained the same when body checking was disallowed [15,25]. The detailed injury analysis results associated with policy change disallowing body checking in non-elite Bantam (13–14 year old) leagues are published elsewhere and support a 56% reduction in injury rate [adjusted incidence rate ratio (IRR): 0.44; (95% CI: 0.27–0.74)] [16].

### 4.2. Strengths and Limitations

Economic evaluation alongside a comparative prospective cohort study provides a unique opportunity to directly identify and measure the healthcare utilization from players as a result of their injuries. The prospective cohort design used in this study capitalized on the variation in body checking policies across Canada to provide an environment for a natural experiment to compare body checking policies and its effect on the rate of injuries and costs. The results may be generalizable to other provinces in Canada since the hockey leagues are similar to the leagues used in this study. This accounts for rules, age categories, and the number of games and practices players are exposed to in a season.

This study does have limitations. The non-randomized nature of this study is a limitation, and although the study design capitalizing on the policy variation across Canada was robust, still the lack of randomization could have resulted in differences between the groups. Some out-of-pocket costs that are related to treatment such as travel costs were not collected and may underestimate the true out of pocket costs from this study. There were some injuries for which there was no reported costs due to missing data on the type of visits or treatments used which leads to underestimate of the total costs. This occurred more frequently in the body checking group thus the cost reduction estimates are conservative. This study took place over two seasons of play, and there is variability between seasons in terms of injuries. Additionally, due to the time horizon of this study, the long-term consequences of injuries and concussions were not measured. As a result, the study findings related to the one year time period likely underestimate the overall healthcare costs associated with hockey-related injuries and on long-term health and quality of life outcomes.

## 5. Conclusions

Players that participate in a league that disallowed body checking had a lower rate of injuries and lower costs to the public healthcare system compared with players in a league that allowed body checking. There is a substantial public health impact both in terms of injuries and costs, and the findings of this study support implementation of policy to disallow body checking in Bantam ice hockey at a national level.

## Figures and Tables

**Table 1:** Healthcare resource types included in each perspective.

Perspective	Healthcare Resource Types
Public healthcare costs	General practitioner/family physician visits, paediatrician visits, sports medicine visits, orthopaedic surgeon visits, emergency department visits (physician billing and technical fees), neurologist, MRI, CT Scan, Ultrasounds, X-rays
Private healthcare costs	Chiropractor visits, physiotherapist visits, massage therapist visits, athletic therapist visits, casts, braces, splints, crutches, over-the-counter and prescribed medication
Total healthcare costs	public healthcare system + private healthcare costs

**Table 2:** Summary of recruitment, game participation hours and game-related injuries.

Outcome	No Body Checking	Body Checking
Number of Teams	33	49
Number of players	396	608
Game participation hours	12,393	23,374
Number of injuries	31	129

**Table 3:** Baseline characteristics of the two study groups.

		No Body Checking	Body Checking
		*n* = 396	*n* = 608
Sex, *n* (%)	Male	390 (98)	597 (98)
	Female	6 (2)	11 (2)
	Missing data	0 (0)	0 (0)
Height, mean (SD) cm		165.7 (10)	164.90 (10)
Missing data, *n* (%)		86 (22)	109 (18)
Weight, mean (SD) kg		55.6 (14)	54.2 (12)
Missing data, *n* (%)		108 (27)	129 (21)
Year of play, *n* (%)	First	195 (49)	329 (54)
	Second	169 (43)	254 (43)
	Missing data	32 (8)	25 (3)
Position, *n* (%)	Forward	195 (49)	325 (54)
	Defence	103 (26)	176 (29)
	Goalie	28 (7)	50 (8)
	Missing data	70 (18)	57 (9)
Previous injury, *n* (%) *	No	276 (70)	432 (71)
	Yes	72 (18)	139 (23)
	Missing data	48 (12)	37 (6)
Previous concussion *n* (%) °	No	252 (64)	403 (66)
	Yes	129 (33)	190 (31)
	Missing data	15 (4)	15 (2)

* Previous concussion or injury 12 months prior to baseline test; ° previous concussion ever.

**Table 4:** Healthcare cost comparison between body checking policy group and by category.

	No Body Checking (*n* = 396)	Body Checking (*n* = 608)
Public healthcare	Costs (2017, $C °)	Costs (2017, $C °)
Visits	$5278	$25,041
Imaging	$2665	$14,188
Casting	$0	$655
Total public healthcare costs	$7943	$39,884
Private healthcare		
Visits	$1015	$2968
Treatments	$240	$435
Medication	$5	$51
Ambulance out-of-pocket fee	$385	
Total private healthcare costs	$1645	$3454
Total public and private healthcare costs	$9588	$43,338

° $C = Canadian dollars.

**Table 5:** Cost-effectiveness analysis results: injury rates and public and private healthcare costs.

	No Body Checking	Body Checking	Difference (No Body Checking Minus Body Checking)
Injury rate (per 1000 player hours) [95% CI] *	2.50 [0.20, 4.80]	5.52 [3.03, 8.01]	−3.02 [−4.01, −1.35]
Base Case: Public Healthcare Perspective			
Cost (per 1000 player-hours) [95% CI]	$641 [$ 266, $ 1095]	$ 1725 [$ 1207, $ 2201]	$ −1084 [$ −1716, $ −416]
Scenario Analysis: Private Healthcare Perspective			
Cost (per 1000 player hours) [95% CI]	$ 102 [$ 25, $ 190]	$ 148 [$ 75, $ 224]	$ −46 [$ −156, $ 70]
Scenario Analysis: Public and Private Healthcare Perspective			
Cost (per 1000 player hours) [95% CI]	$ 774 [$ 332, $ 1308]	$ 1874 [$ 1324, $ 2412]	−$ 1100 [$ −1804, $ −346]

* Lower and upper 95% confidence intervals for the injury and costs rates for each group were based on bootstrapped 2.5 and 97.5 percentiles.

**Table 6:** Provincial and national cost difference projections (no body checking—body checking).

Alberta Projection *	
Public healthcare costs	$ −187,364 (95% CI $ −296,617, $ −71,903)
Private healthcare costs	$ −7972 (95% CI $ −26,965, $12,037)
Total healthcare costs	$ −190,188 (95% CI $ −311,798, $ −59,761)
Canadian Projection *	
Public healthcare costs	$ −1,602,397 (95% CI $ −2,536,769, $ −614,936)
Private healthcare costs	$ −68,183 (95% CI $ −230,614, $102,940)
Total healthcare costs	$ −1,626,554 (95% CI $ −2,666,602, $ −511,084)

* The average player-hours estimate is 38.75 h per Bantam player. The population of Bantam players in Alberta 2016–2017 season was 7435, so the non-elite population (lower 60%) was 4461, and population in Canada was 63,587, so the non-elite (lower 60%) population was 38,152.

## Data Availability

The data presented in this study are available on request from the corresponding author. The data are not publicly available due to provisions of the ethics approval.
