# Correction: Currie et al. Reality Check 2: The Cost-Effectiveness of Policy Disallowing Body Checking in Non-Elite 13- to 14-Year-Old Ice Hockey Players. *Int. J. Environ. Res. Public Health* 2021, *18*, 6322

**DOI:** 10.3390/ijerph21030269

**Published:** 2024-02-27

**Authors:** Gillian R. Currie, Raymond Lee, Luz Palacios-Derflingher, Brent Hagel, Amanda M. Black, Shelina Babul, Martin Mrazik, Deborah A. Marshall, Carolyn A. Emery

**Affiliations:** 1 Department of Paediatrics, Cumming School of Medicine, University of Calgary, Calgary, AB T3B 6A8, Canada; brent.hagel@ahs.ca (B.H.); caemery@ucalgary.ca (C.A.E.); 2 Department of Community Health Sciences, Cumming School of Medicine, University of Calgary, Calgary, AB T2N 4Z6, Canada; raymond.lee47@gmail.com (R.L.); damarsha@ucalgary.ca (D.A.M.); 3 Sport Injury Prevention Research Centre, Faculty of Kinesiology, University of Calgary, Calgary, AB T2N 1N4, Canada; lmpalaci@ucalgary.ca (L.P.-D.); ablack@ucalgary.ca (A.M.B.); 4 Department of Pediatrics, University of British Columbia, Vancouver, BC V6H 3V4, Canada; sbabul@bcchr.ca; 5 BCIRPU, BC Children’s Hospital, Vancouver, BC V6H 3V4, Canada; 6 Department of Educational Psychology, University of Alberta, Edmonton, AB T6G 2G5, Canada; mrazik@ualberta.ca; 7 McCaig Bone and Joint Health Institute, Calgary, AB T2N 4Z6, Canada

There was an error in our original publication [1]. We identified an error in the coding of exposure data, which affected our estimates of rates of injury and costs per 1000 player-hours reported in the original paper. The authors state that the scientific conclusions are unaffected. This correction was approved by the Academic Editor. The original publication has also been updated.

## Errors in Figures and Tables

In the original publication, there was a mistake in Table 2 (in Section 3 Results) as published. The game participation hours were incorrect for both groups. The corrected version of Table 2 appears below.

**Table 2:** Summary of recruitment, game participation hours and game-related injuries.

Outcome	No Body Checking	Body Checking
Number of Teams	33	49
Number of players	396	608
Game participation hours	12,393	23,374
Number of injuries	31	129

In the original publication, there was a mistake in Table 5 (in Section 3 Results) as published. Due to the correction to participation hours in Table 2, the reported rates per 1000 player hours changed. The corrected version of Table 5 appears below.

**Table 5:** Cost-effectiveness analysis results: injury rates and public and private healthcare costs.

	No Body Checking	Body Checking	Difference (No Body Checking Minus Body Checking)
Injury rate (per 1000 player hours) [95% CI] *	2.50 [0.20, 4.80]	5.52 [3.03, 8.01]	−3.02 [−4.01, −1.35]
Base Case: Public Healthcare Perspective			
Cost (per 1000 player-hours) [95% CI]	$641 [$ 266, $ 1095]	$ 1725 [$ 1207, $ 2201]	$ −1084 [$ −1716, $ −416]
Scenario Analysis: Private Healthcare Perspective			
Cost (per 1000 player hours) [95% CI]	$ 102 [$ 25, $ 190]	$ 148 [$ 75, $ 224]	$ −46 [$ −156, $ 70]
Scenario Analysis: Public and Private Healthcare Perspective			
Cost (per 1000 player hours) [95% CI]	$ 774 [$ 332, $ 1308]	$ 1874 [$ 1324, $ 2412]	−$ 1100 [$ −1804, $ −346]

* Lower and upper 95% confidence intervals for the injury and costs rates for each group were based on bootstrapped 2.5 and 97.5 percentiles.

In the original publication, there was a mistake in Table 6 (in Section 3 Results) as published. Due to the correction to participation hours in Table 2, the reported rates per 1000 player hours changed in Table 5, which also affected the projections in Table 6. The corrected version of Table 6 appears below.

**Table 6:** Provincial and national cost difference projections (no body checking—body checking).

Alberta Projection *	
Public healthcare costs	$ −187,364 (95% CI $ −296,617, $ −71,903)
Private healthcare costs	$ −7972 (95% CI $ −26,965, $12,037)
Total healthcare costs	$ −190,188 (95% CI $ −311,798, $ −59,761)
Canadian Projection *	
Public healthcare costs	$ −1,602,397 (95% CI $ −2,536,769, $ −614,936)
Private healthcare costs	$ −68,183 (95% CI $ −230,614, $102,940)
Total healthcare costs	$ −1,626,554 (95% CI $ −2,666,602, $ −511,084)

* The average player-hours estimate is 38.75 h per Bantam player. The population of Bantam players in Alberta 2016–2017 season was 7435, so the non-elite population (lower 60%) was 4461, and population in Canada was 63,587, so the non-elite (lower 60%) population was 38,152.

In the original publication, there was a mistake in Figure 1 (in Section 3 Results) as published. Due to the correction to participation hours in Table 2, the reported rates per 1000 player hours changed in Table 5, which also affected Figure 1. The corrected version of Figure 1 appears below.

**Figure 1:**
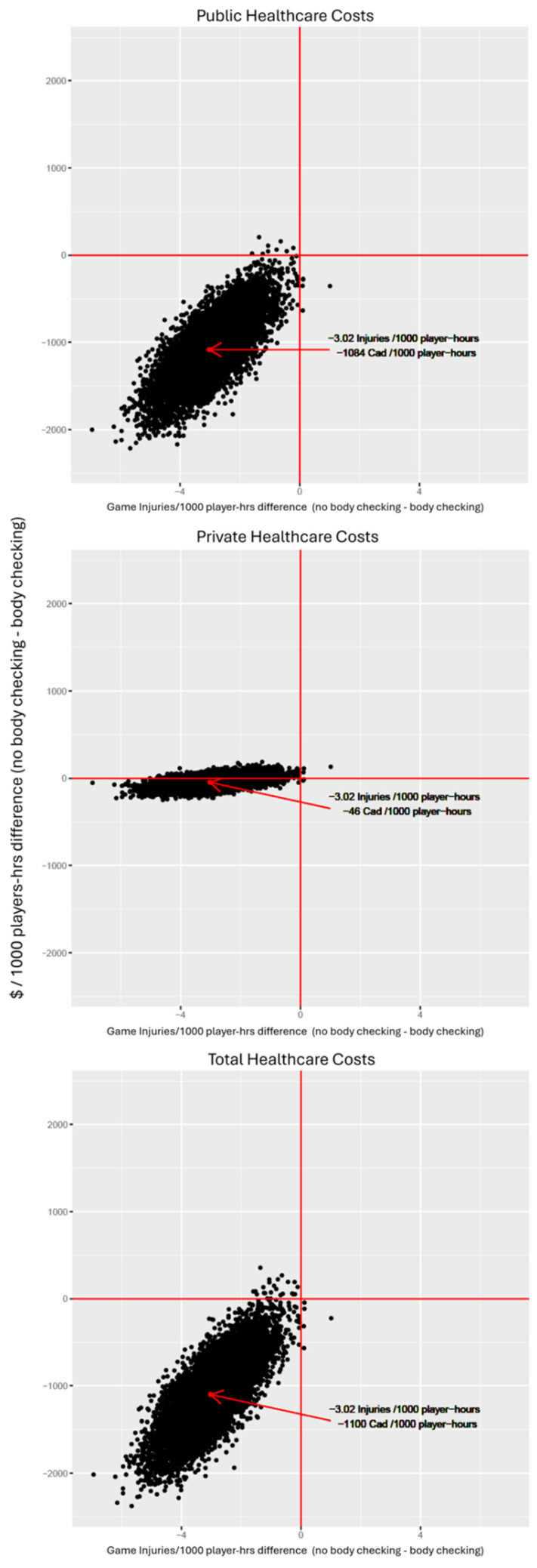
Probabilistic sensitivity analysis for policy comparison of body checking to no body checking.

## Text Correction

The error noted above which affected the numbers in the tables also appeared in the text in multiple locations as follows:

1. A correction has been made to the Abstract. The corrected abstract appears below.

**Abstract:** Sport-related injuries are the leading cause of injury in youth and are costly to the healthcare system. When body checking is disallowed in non-elite levels of Bantam (ages 13–14 years) ice hockey, the injury rate is reduced, however the impact on costs is unknown. This study compared rates of game injuries and costs among non-elite Bantam ice hockey leagues that disallow body checking to those that did not. Methods: An economic evaluation was conducted alongside a prospective cohort study comparing 608 players from leagues where body checking was allowed in games (Calgary/Edmonton 2014–2015, Edmonton 2015–2016) with 396 players from leagues where it was not allowed in games (Vancouver, Kelowna 2014–2015, Calgary in 2015–2016). The effectiveness measure was the rate of game injuries per 1000 player-hours. Costs were estimated based on associated healthcare use within the publicly funded healthcare system as well as privately paid healthcare costs. Probabilistic sensitivity analysis was conducted using bootstrapping. Results: Disallowing body checking reduced the rate of injuries by 3.02 per 1000 player hours (95% CI −4.01, −1.35) and reduced public and total healthcare system costs by $ 1084 (95% CI $ −1716, $ −416) and $ 1100 (95% CI $ −1804, $ −346 per 1000 player-hours, respectively. These findings were robust in over 99% of iterations in sensitivity analyses in the public healthcare and the total healthcare system perspectives. There was no statistically significant difference in privately paid healthcare costs ($ −46 per 1000 player hours (95% CI $ −156, $ 70)). Interpretation: Disallowing body checking in non-elite 13–14-year-old ice hockey nationally would prevent injuries and reduce public healthcare costs.

2. A correction has been made to the Introduction section, second paragraph. The corrected sentence appears below.

A similar policy in Bantam (age 13–14) hockey also reduced injury rates by 55% [17]; however, the effect on costs has not been studied.

3. A correction has been made to the Results section, first paragraph. The corrected sentence appears below.

As previously reported [17], a total of 82 teams comprising 944 unique players were recruited in the study in the 2014–2015 and 2015–2016 seasons; 60 players participated in both seasons. There were 33 teams that were disallowed from body checking and 49 teams that were allowed to body check. As shown in Table 2, players disallowed from body checking had a total of 12,393 game participation hours in 396 players and 23,374 participation hours were observed in 608 players who were allowed to body check. When body checking was allowed, 129 injuries occurred compared with 31 injuries when body checking was disallowed. Table 3 shows that the distribution of baseline characteristics (sex, weight, height, player position, previous injury over the last year, previous concussion) was similar between the two groups [17].

4. A correction has been made to the Results section, Section 3.1. Cost-Effectiveness Analysis Results, first and second paragraph. The corrected first paragraph and the corrected first sentence of the second paragraph appear below.

As shown in Table 5, for the base-case analysis using the public healthcare system perspective, there is a reduction in the rate of injuries by 3.02 per 1000 player hours (95% CI –4.01, −1.35) and a reduction in the costs by $ 1084 per 1000 player hours (95% CI $ −1716, $ −416) when body checking was disallowed compared with when it is allowed. There was no statistically significant difference in private healthcare costs ($ −46 per 1000 player hours (95% CI $ −156, $ 70)). Considering the total healthcare cost perspective, there was a reduction in costs by $ 1100 per 1000 player hours (95% CI $ −1804, $ −346).

In probabilistic sensitivity analysis (Figure 1), the no body checking policy reduces both injuries and costs from the public and total healthcare perspectives in 99.9% and 99.5% of iterations, respectively.

5. A correction has been made to the Results section, Section 3.2. Provincial and National Projection Results, first paragraph. The corrected text appears below.

As previously reported [17], if a policy disallowing body checking had been adopted in the 2015–2016 season, 522 injuries could have been prevented (95% CI −694, −233) in Alberta and 4461 injuries could have been prevented in Canada (95% CI −5932, −1994). In the current study, we similarly apply the cost rate reductions to the provincial and national player populations (see Table 6) to estimate a total potential savings of $ 187,364 public healthcare costs in Alberta (95% CI $ −296,617, $ −71,903) and $ 1,602,397 public healthcare costs in Canada (95% CI $ −2,536,769, $ −614,936). The results for the private healthcare cost and total healthcare cost perspective are presented in Table 6.

6. A correction has been made to the Discussion section, Section 4.1. Interpretation, first paragraph. The corrected text appears below.

These study findings support a policy recommendation to disallow body checking in games in 13- to 14-year-old ice hockey leagues as the policy is associated with both reduced rate of game-related injuries and reduced public healthcare costs. The findings were robust in sensitivity analysis. Projecting the results to all Bantam players in Alberta and Canada in the 2015–2016 hockey season, disallowing body checking in non-elite Bantam players could prevent 522 injuries and save $ 187,364 in public healthcare costs in Alberta. At a national level, this could prevent 4461 injuries and save $ 1,602,397 in public healthcare costs. In the context of total public healthcare spending in Alberta and in Canada (that is $ 21 and $ 161 billion, respectively in 2015 [24]), this is a small proportion; however, it is still an expenditure that could be saved, as well as avoiding injuries for these children. This study looks at injuries and costs over the one year of the study, so does not account for the longer-term impacts of those injuries.

7. A correction has been made to the Acknowledgments section. The corrected text appears below.
